# Supplementary material for: Scalable Production and In Vitro Efficacy of Inhaled Erlotinib Nanoemulsion for Enhanced Efficacy in Non-Small Cell Lung Cancer (NSCLC)
Source: Pharmaceutics. 2023 Mar 20;15(3):996. doi: 10.3390/pharmaceutics15030996 (PMC10054254; doi:10.3390/pharmaceutics15030996)
Supplement: Supplementary file 1 [file pharmaceutics-15-00996-s001.zip › pharmaceutics-2224588-supplementary.pdf]

<u>SUPPLEMENTARY INFORMATION</u>

Scalable Production and *In-vitro* Efficacy of Inhaled Erlotinib Nano-emulsion for Enhanced Efficacy in Resistant NSCLC

Gautam Chauhan[1], Xuechun Wang[1], Carol Yousry[1,2], Vivek Gupta[1,*]

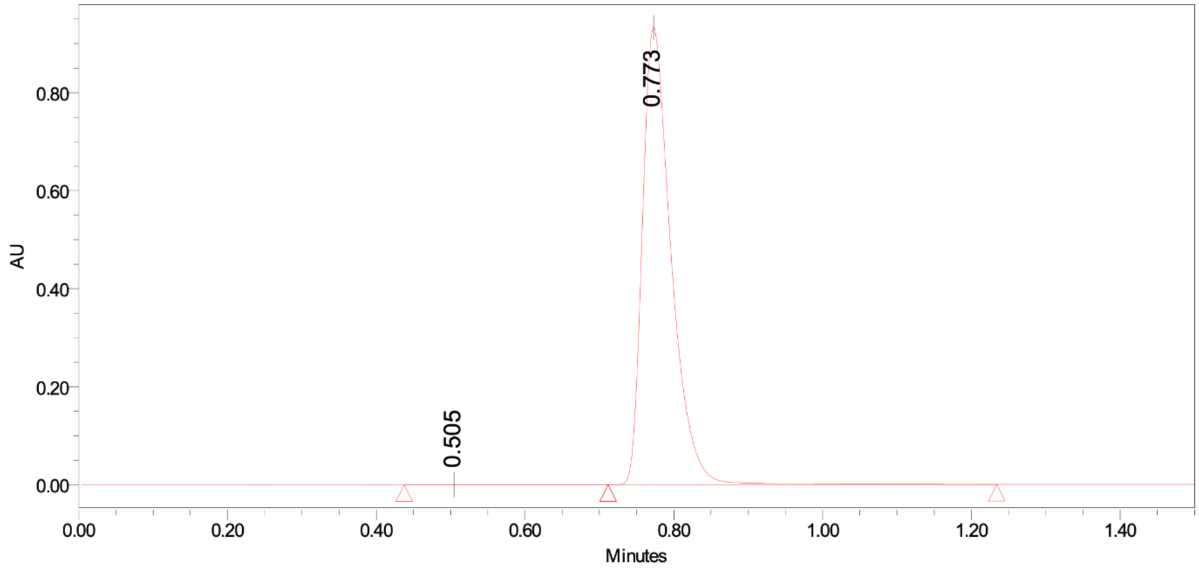

Figure S1: A representative UPLC chromatogram for Erlotinib quantification.
